# Altered Body Weight Regulation in* CK1ε* Null and* tau* Mutant Mice on Regular Chow and High Fat Diets

**DOI:** 10.1155/2016/4973242

**Published:** 2016-04-06

**Authors:** Lili Zhou, Keith C. Summa, Christopher Olker, Martha H. Vitaterna, Fred W. Turek

**Affiliations:** ^1^Center for Sleep and Circadian Biology, Northwestern University, Evanston, IL 60208, USA; ^2^Department of Neurobiology, Northwestern University, Evanston, IL 60208, USA

## Abstract

Disruption of circadian rhythms results in metabolic dysfunction. Casein kinase 1 epsilon (*CK1ε*) is a canonical circadian clock gene. Null and* tau* mutations in* CK1ε* show distinct effects on circadian period. To investigate the role of* CK1ε* in body weight regulation under both regular chow (RC) and high fat (HF) diet conditions, we examined body weight on both RC and HF diets in* CK1ε* ^−/−^ and* CK1ε* ^*tau*/*tau*^ mice on a standard 24 hr light-dark (LD) cycle. Given the abnormal entrainment of* CK1ε* ^*tau*/*tau*^ mice on a 24 hr LD cycle, a separate set of* CK1ε* ^*tau*/*tau*^ mice were tested under both diet conditions on a 20 hr LD cycle, which more closely matches their endogenous period length. On the RC diet, both* CK1ε* ^−/−^ and* CK1ε* ^*tau*/*tau*^ mutants on a 24 hr LD cycle and* CK1ε* ^*tau*/*tau*^ mice on a 20 hr LD cycle exhibited significantly lower body weights, despite similar overall food intake and activity levels. On the HF diet,* CK1ε* ^*tau*/*tau*^ mice on a 20 hr LD cycle were protected against the development of HF diet-induced excess weight gain. These results provide additional evidence supporting a link between circadian rhythms and energy regulation at the genetic level, particularly highlighting* CK1ε* involved in the integration of circadian biology and metabolic physiology.

## 1. Introduction

The coordination of daily rhythms in feeding behavior, body temperature, and energy storage and utilization across the 24 hr light-dark (LD) cycle is critical in maintaining homeostasis. It has been well established that circadian rhythms are controlled by endogenous circadian clock genes [1]. Core clock genes, such as* Clock*,* Bmal1*,* Period* (*Per*), and* Cryptochrome* (*Cry*), are key components of the transcriptional/translational feedback loop, which is considered to be the major mechanism underlying the generation of circadian rhythms [2]. Based on gene expression profiling studies, approximately 3%–20% of the transcriptome in any given tissue exhibits circadian oscillation, and a large proportion of the rhythmically regulated transcripts are involved in metabolic function [3]. In addition, the discovery that clock genes function in peripheral tissues involved in metabolism, such as liver, fat, heart, and muscle, provides further support that circadian and metabolic processes are tightly linked [4, 5].

Accumulating evidence from both human and animal studies has strongly supported an important role for circadian rhythms in the regulation of metabolism. Shift workers have a higher incidence of diabetes, obesity, cancer, and cardiovascular diseases [6–8]. In clinical studies, forced misalignment of behavioral (i.e., sleep/wake) and circadian cycles in human subjects causes metabolic and endocrine abnormalities, including decreases in leptin and increases in glucose and insulin [9]. In rodents, genetic disruption of the molecular clock system leads to a variety of metabolic abnormalities, including obesity and metabolic syndrome in* Clock* mutant mice [10] and* Per2* deficient mice [11]. In contrast, mutant mice lacking the core clock gene* Bmal1*, either globally or exclusively in liver, have a lean phenotype [5, 12, 13]. Chronic circadian disruption, achieved by housing wild-type mice in a 20 hr LD cycle that is incongruous with their endogenous ~24 hr circadian period, results in accelerated weight gain and obesity, as well as dysregulation of metabolic hormones [14].

Casein kinase 1 epsilon (*CK1ε*), a member of the serine/threonine protein kinase family, is a canonical circadian gene which regulates circadian rhythms through posttranslational modification of the PER and CRY proteins [15]. In the kinase domain of* CK1ε*, a C to T single nucleotide transition results in a missense point mutation (*tau* mutation) of a conserved amino acid residue 178 (R178C), which causes profound changes in circadian organization in hamsters and mice, with a 4-hour decrease in the free-running period in homozygous mutants in constant darkness (DD) [16, 17]. Furthermore, the intrinsic 20 hr period length in* tau* mutant mice prevents stable entrainment to a conventional 24 hr LD cycle [15]. Another mouse model for* CK1ε* is the null mutant, which was developed using the loxP-Cre strategy to create a premature codon induced by frameshift in the* CK1ε* gene. In contrast to* tau* mutant mice, null mutant mice exhibited a significant but very mild lengthening of the circadian period [18].

Despite extensive evidence supporting a tight relationship between circadian rhythms and metabolism, studies examining the role of* CK1ε* in metabolic function remain limited. Previous studies in hamsters have demonstrated a significant impact of the* CK1ε tau* mutation on body weight regulation in males, with homozygous mutants weighing significantly less than wild-types [19–21]. Both* tau* mutant hamsters and* tau* mutant mice exhibit increased metabolic rates [18, 20]. Despite these findings, it is unknown whether* tau* mutant mice exhibit a reduction in body weight. In addition, no previous study has examined other mutations in* CK1ε*; thus, no comparison between different* CK1ε* mutants has been made. Furthermore, it is unclear whether the metabolic changes in* CK1ε tau* mutant hamsters are caused by an accelerated circadian pacemaker, as opposed to other pleiotropic effects of the mutant allele. It is also unknown whether* tau* mutants demonstrate altered body weight regulation on a high fat diet (HF) as they do on regular chow (RC).

Therefore, we used both* CK1ε* ^−/−^ and* CK1ε* ^*tau*/*tau*^ mutant mice as genetic tools to investigate the impact of these two mutations on body weight regulation in a 24 hr LD cycle under two different diet conditions: RC and HF. In addition, because of the abnormal entrainment in* CK1ε* ^*tau*/*tau*^ mice (not* CK1ε* ^−/−^ mice) on a 24 hr LD cycle, a separate set of* CK1ε* ^*tau*/*tau*^ mice were tested in a 20 hr LD cycle, which more closely matches their endogenous period length and permits patterns of entrainment comparable to those of wild-types on a standard 24 hr LD cycle. This was done to investigate whether the phenotype observed in* CK1ε* ^*tau*/*tau*^ mice on a 24 hr LD cycle was simply an artifact of the altered entrainment. Our results indicate that, on a RC diet, both* CK1ε* null and* tau* mutations on a 24 hr LD cycle, as well as the* CK1ε tau* mutation on a 20 hr LD cycle, exhibit significant effects on body weight, with mutant mice weighing less than wild-types. In contrast, on the HF diet, neither mutation on a 24 hr LD cycle led to a significant difference from wild-types. Remarkably, a 20 hr LD cycle, which restores normal light entrainment in* CK1ε* ^*tau*/*tau*^ mice, provides resistance to excess body weight gain induced by a HF diet.

## 2. Materials and Methods

### 2.1. Animals and Experimental Protocol

All mutant animals used in this experiment were coisogenic C57BL/6J mice. The generation of these mutants has been described previously [18]. For all experiments, male wild-type (*CK1ε* ^+/+^),* CK1ε* null mutant (*CK1ε* ^−/−^), and* tau* mutant (*CK1ε* ^*tau*/*tau*^) mice were maintained in standard mouse cages with food and water available* ad libitum* on a conventional 12 hr light : 12 hr dark LD cycle (LD 12 : 12; lights on at 0600, lights off at 1800; 300 lux and 22–24°C ambient temperature). Until the beginning of the study, mice were group-housed and fed a RC diet (16% kcal from fat, 27% kcal from protein, and 57% kcal from carbohydrate; 7012 Teklad LM-485 Mouse/Rat Sterilizable Diet, Harlan Laboratories, Inc., Indianapolis, IN). At the age of 10 weeks, mice were transferred to individual cages under the same lighting and environmental conditions described above. The animals were randomized into experimental groups and fed either RC (*CK1ε* ^*+/+*^, *n* = 18;* CK1ε* ^−/−^, *n* = 17;* CK1ε* ^*tau*/*tau*^, *n* = 13) or HF diet (45% kcal from fat, 20% kcal from protein, and 35% kcal from carbohydrate; D12451, Research Diets, Inc. New Brunswick, NJ;* CK1ε* ^*+/+*^, *n* = 16;* CK1ε* ^−/−^, *n* = 13;* CK1ε* ^*tau*/*tau*^, *n* = 16).

Body weight was recorded weekly for 6 weeks. Food consumption was measured daily for 7 consecutive days in the second week of the experiment. A glucose tolerance test and an insulin tolerance test were performed on the seventh and eighth weeks, respectively, as described below. On the ninth week, serum samples, obtained by tail bleed, were collected 6 hours after light onset (by convention, referred to as Zeitgeber time (ZT) 6) from mice that had been fasted for 6 hours. At the end of the experiment, mice were euthanized (without fasting) at ZT6, and gonadal fat pads were harvested for analysis. A separate group of age-matched* CK1ε* ^*tau*/*tau*^ mutant mice were individually housed and fed either RC (*n* = 10) or HF (*n* = 8) diet. All procedures for this group were the same as above, except they were maintained on a 20 hr LD cycle (LD 10 : 10) for the duration of the experimental protocol. All procedures and protocols were approved in advance by the Institutional Animal Care and Use Committee of Northwestern University.

### 2.2. Locomotor Activity

Five or six mice of each genotype from a separate group of mice were singly housed in individual cages outfitted for locomotor activity analysis via detection of infrared beam breaks. These mice were fed either RC or HF diet under LD 12 : 12 or LD 10 : 10 cycle for 10 days and were used exclusively for locomotor activity analysis (i.e., they were not included in the body weight measurements or other metabolic analyses). Beam breaks were recorded in 6 min bins using the Chronobiology Kit (Stanford Software Systems, Stanford, CA, USA) and were analyzed using the ClockLab software (Actimetrics, Wilmette, IL, USA).

### 2.3. Body Temperature

Body temperatures were measured in 8–16-week-old male mice with a rectal thermometer (4600 thermometer, Measurement Specialties, Beavercreek, OH) inserted 1.7 cm in the middle of day time.

### 2.4. Blood Collection and Serum Insulin Analysis

Mice were transferred to clean cages at ZT0 to fast for 6 hours, during which access to water was unrestricted. At ZT6, tails were clipped to obtain blood samples for analysis and serum isolation. Briefly, a small (1 mm) cut was made at the end of the tail and about 50 *μ*L of blood was obtained by gentle massage using tail blood collection tubes (BD Vacutainer Plus plastic serum tube, 2 mL, red top, #367820, BD Diagnostics, Franklin Lakes, NJ). Mice were then returned to their home cages. At room temperature, blood was incubated in an upright position for 45 min and then spun in a centrifuge for 6 min at 2000 ×g. The supernatant (serum) was removed, frozen, and stored at −80°C until future analysis. Serum insulin levels were determined by ELISA (Ultra Sensitive Mouse Insulin ELISA Kits, Crystal Chem Inc., Downers Grove, IL) according to the manufacturer's instructions. Blood glucose was determined as described below.

### 2.5. Glucose and Insulin Tolerance Tests

For the glucose tolerance test (GTT), mice were fasted for 6 hours (ZT0 to ZT6), after which baseline blood glucose levels were determined from a blood sample obtained from the tail of each mouse. A small (1 mm) cut was made at the end of the tail and a drop of blood was deposited onto a glucometer strip (Abbott Laboratories, Abbott Park, IL) by gentle massage for assessment of blood glucose level. Mice were then immediately injected intraperitoneally with 1.0 g/kg body weight of glucose (G8769, Sigma-Aldrich, St. Louis, MO). Additional blood samples and blood glucose readings were obtained via massage of the tail nick at 30, 60, and 120 min after the injection. Upon completion of the experiment, mice were returned to their home cages. Results are expressed as percentage of baseline glucose. Area under the curve (AUC) values were calculated by the trapezoidal rule.

For the insulin tolerance test (ITT), mice were fasted for 2 hours (ZT4 to ZT6), after which baseline blood glucose was measured as described above. Immediately after the baseline blood glucose sample was obtained, 0.75 units/kg body weight of regular human insulin Humulin® R U-100 (Eli Lilly, Indianapolis, IN; insulin was diluted to 1 : 1000 (0.1 units/mL) with sterile diluent) was injected intraperitoneally. After the insulin injection, blood glucose was sampled at 30, 60, 90, and 120 min, as described above. Upon completion of the experiment, animals were returned to their home cages. Results are expressed as percentage of baseline glucose. Area under the curve (AUC) values were calculated by the trapezoidal rule.

### 2.6. Statistics

All statistical analysis was performed using R software (http://www.r-project.org/) [22]. Time course data of body weight, GTT, and ITT on each diet were analyzed using repeated measures ANOVA with genotype as the between-subject factor and time as the within-subject variable. Following a significant result on repeated measures ANOVA, single time point comparisons were made by Benjamini-Hochberg multiple comparison tests. All the other comparisons between genotypes and diets were conducted via two-way ANOVA, with Benjamini-Hochberg post hoc tests performed where appropriate for multiple comparisons. Group values are expressed as mean ± SEM. Significant differences were defined as *p* < 0.05.

## 3. Results

### 3.1. Altered Body Weight Regulation in* CK1ε* Mutants

**Figure 1:**
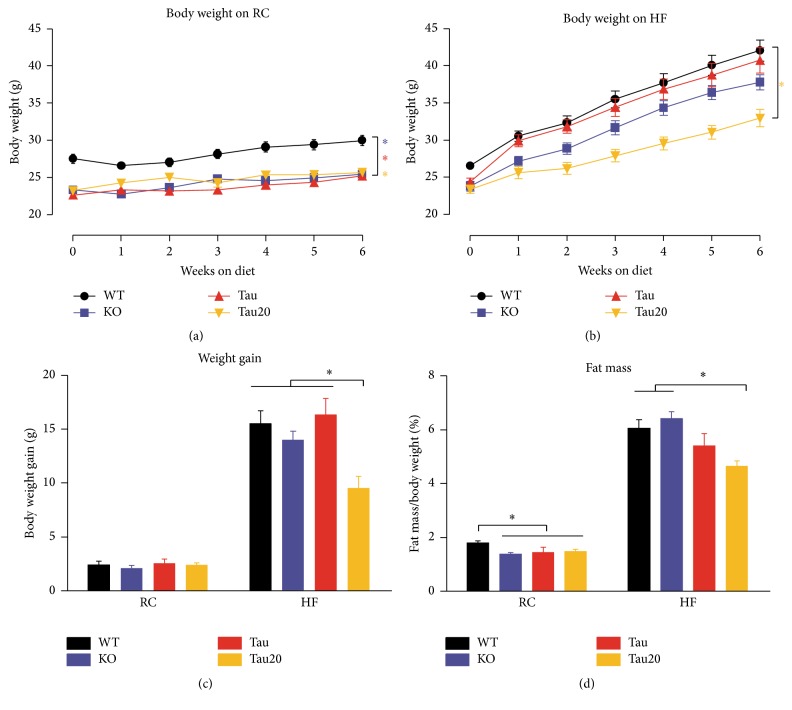
Altered body weight and fat mass in* CK1ε* mutant mice. (a) Growth curves of mice on RC diet. (b) Growth curves of mice on HF diet. (c) Total body weight gain after six weeks of either RC or HF diet. Body weight gain was calculated by subtracting the baseline weight at the beginning of the diet experiment from weight at the end of the diet experiment. (d) The gonadal fat pads from mice fed either RC or HF diet were weighed at the end of study.* CK1ε* ^*+/+*^ (black),* CK1ε* ^−/−^ (blue), and* CK1ε* ^*tau*/*tau*^ (red) mice were on a 24 hr LD cycle;* CK1ε* ^*tau*/*tau*^ mice kept on a 20 hr LD cycle are represented in yellow. Mean values are presented for each group, with error bars representing SEM. Asterisks indicate significant differences between groups (*p* < 0.05).

Individually caged young adult (10-week) male mice were given either regular chow (RC) or high fat (HF) diet for the entire experimental protocol. After 6 weeks on RC, both* CK1ε* ^−/−^ and* CK1ε* ^*tau*/*tau*^ mice exhibited a significantly lower body weight than* CK1ε* ^*+/+*^ mice, approximately 15% less (Figure 1(a);* CK1ε* ^+/+^ = 29.38 ± 1.39 g,* CK1ε* ^−/−^ = 24.91 ± 0.98 g, and* CK1ε* ^*tau*/*tau*^ = 24.69 ± 1.67 g). Because the body weight of* CK1ε* ^+/−^ and* CK1ε* ^+/*tau*^ mice did not differ from wild-type mice (data not shown), the present study only focuses on results from homozygous mutants. Additional analyses indicated that the body weight differences between* CK1ε* ^*+/+*^ mice and both* CK1ε* mutant mice were as early as the age of 3 weeks immediately after weaning (see Figure S1 in Supplementary Material available online at http://dx.doi.org/10.1155/2016/4973242).* CK1ε* ^−/−^ mice had significantly lower body weight than wild-type mice throughout the entire period of experiment. However, the stable body weight changes of* CK1ε* ^*tau*/*tau*^ mice compared to wild-type mice were only observed after week 8. To avoid this developmental fluctuation, we focused on the body weight only during adulthood.

Due to abnormal entrainment patterns and the mismatch between endogenous period length and the 24 hr LD cycle in homozygous* tau* mutants [15], we were interested in whether restoration of entrainment and resonance between the environmental cycle length and endogenous period in* tau* mutants would impact body weight regulation.* CK1ε* ^−/−^ mice, whose endogenous period is very close to 24 hr, have normal entrainment, so their phenotype was less likely to be affected by the entrainment. Therefore, we focused on only* CK1ε* ^*tau*/*tau*^ mice and maintained two separate groups of* tau* mutant mice on RC and HF diet, respectively, on a 20 hr LD cycle. We observed that the* CK1ε* ^*tau*/*tau*^ mice on a 20 hr LD cycle maintain significantly reduced body weight compared to wild-type mice on a 24 hr LD cycle on RC diet (Figure 1(a)).

On the HF diet, mice of all genotypes gained significantly more weight than the ones on the RC diet, as expected (Figure 1(c)). Significant differences were not evident between mutant and wild-type mice on a 24 hr LD cycle (Figure 1(b)). Intriguingly, when HF-fed* CK1ε* ^*tau*/*tau*^ mice were housed on a 20 hr LD cycle, the rate of body weight gain was reduced compared to other genotypes, resulting in a significantly lower weight gain after 6 weeks on the diet (Figure 1(c);* CK1ε* ^+/+^ = 15.5 ± 1.2 g,* CK1ε* ^−/−^ = 14.0 ± 0.8 g,* CK1ε* ^*tau*/*tau*^ = 16.4 ± 1.4 g, and* CK1ε* ^*tau*/*tau*^ on 20 hr LD = 9.6 ± 1.1 g).

In agreement with the observed effects on body weight, we also observed reduced gonadal fat pad weight in* CK1ε* mutant mice. As shown in Figure 1(d), on RC, both* CK1ε* ^−/−^ and* CK1ε* ^*tau*/*tau*^ mutant mice on a 24 hr LD cycle, as well as* CK1ε* ^*tau*/*tau*^ on a 20 hr LD cycle, had a significantly reduced proportion of gonadal fat pad mass to total body weight (*CK1ε* ^*+/+*^ = 1.79 ± 0.09%,* CK1ε* ^−/−^ = 1.37 ± 0.05%,* CK1ε* ^*tau*/*tau*^ = 1.44 ± 0.19%, and* CK1ε* ^*tau*/*tau*^ on 20 hr LD = 1.48 ± 0.07%). On the HF diet (Figure 1(d)), mice of all genotypes exhibited a pronounced increase in percentage of gonadal fat pad weight, as expected; however, no significant differences were observed between* CK1ε* ^−/−^,* CK1ε* ^*tau*/*tau*^, and* CK1ε* ^*+/+*^ mice on a 24 hr LD cycle. Interestingly, as with body weight, HF-fed* CK1ε* ^*tau*/*tau*^ mutant mice on a 20 hr LD cycle had a significant reduction in the percentage of gonadal fat pad weight, compared to HF-fed wild-type mice (*CK1ε* ^*+/+*^ = 6.05 ± 0.32% and* CK1ε* ^*tau*/*tau*^ on 20 hr LD = 4.64 ± 0.21%). With respect to the absolute mass of the gonadal fat pad, identical results were observed (data not shown).

### 3.2. Altered Diurnal Feeding Behavior and Locomotor Activity in* CK1ε* Mutants

**Figure 2:**
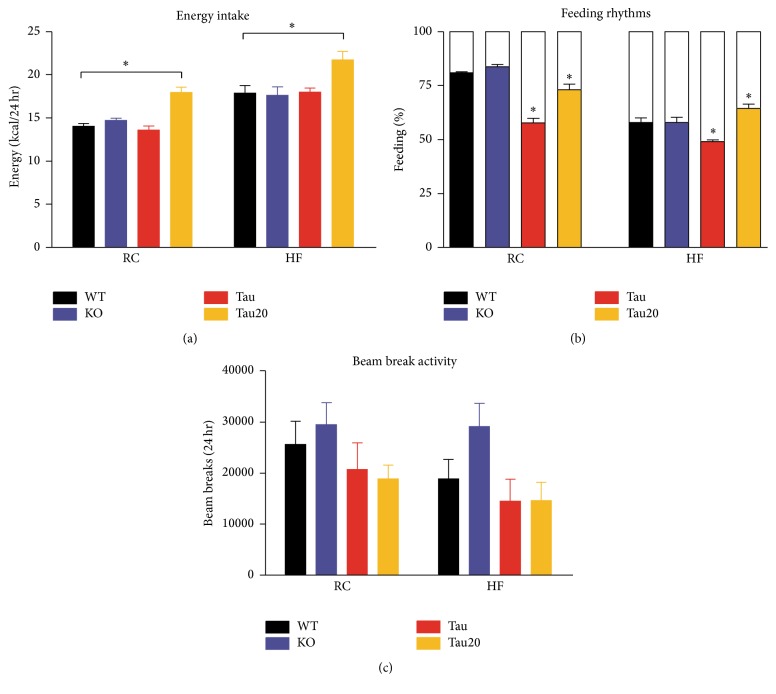
Altered diurnal feeding behaviors in* CK1ε* mutant mice. (a) The diet, but not the genotype, has a significant effect on daily calorie intake. Energy intake was expressed as the average kilocalories consumed during each 24 hr period. (b) Percentage of calorie intake in light and dark periods. The top proportion, in white, represents the percentage of calorie intake in the light period. The bottom proportion, in different colors, represents the percentage of calorie intake in the dark period. Results were compared between mutants and wild-type controls on the same diet. (c) Locomotor activity of mice fed either RC or HF diet. The activity was expressed as the average beam breaks during each 24 hr period.* CK1ε* ^*+/+*^ (black),* CK1ε* ^−/−^ (blue), and* CK1ε* ^*tau*/*tau*^ (red) mice were on a 24 hr LD cycle;* CK1ε* ^*tau*/*tau*^ mice represented in yellow were on a 20 hr LD cycle. Mean values are presented for each group, with error bars representing SEM. Asterisks indicate significant differences between groups (*p* < 0.05).

To determine whether the reduced body weight in* CK1ε* mutants was due to decreased food intake, we examined food consumption under RC and HF conditions. Total daily overall caloric intake did not differ between genotypes on 24 hr LD cycle (Figure 2(a)). However,* CK1ε* ^*tau*/*tau*^ mice on a 20 hr LD cycle consumed more energy than wild-type mice every 24 hr (RC:* CK1ε* ^*+/+*^ = 14.0 ± 0.3 kcal,* CK1ε* ^*tau*/*tau*^ on 20 hr LD = 17.9 ± 0.6 kcal; HF:* CK1ε* ^*+/+*^ = 17.9 ± 0.9 kcal,* CK1ε* ^*tau*/*tau*^ on 20 hr LD = 21.7 ± 1.0%). The distribution of food intake during the light versus dark periods was altered in the* CK1ε* mutants compared to wild-types (Figure 2(b)). On RC,* CK1ε* ^−/−^ mice consumed a greater proportion of their total daily calories during the dark period. In contrast,* CK1ε* ^*tau*/*tau*^ mice consumed much less diet during the dark period (Figure 2(b);* CK1ε* ^*+/+*^ = 80.9 ± 1.0%,* CK1ε* ^−/−^ = 83.8 ± 1.0%, and* CK1ε* ^*tau*/*tau*^ = 57.8 ± 2.2%). Thus, the diurnal rhythm in energy intake in* CK1ε* ^*tau*/*tau*^ mice was greatly attenuated on a 24 hr LD cycle. Interestingly, RC-fed* CK1ε* ^*tau*/*tau*^ mice housed on a 20 hr LD cycle consumed 73.1% of their total daily calories during dark period. This remained significantly lower than that of wild-type control mice but was improved compared to that of* CK1ε* ^*tau*/*tau*^ mice on a 24 hr LD cycle (Figure 2(b)). On the HF diet, diurnal rhythms of food intake in mice of all three genotypes on a 24 hr LD cycle were attenuated (Figure 2(b)). In particular,* CK1ε* ^*tau*/*tau*^ mice on a 24 hr LD cycle exhibited the greatest attenuation of diurnal feeding rhythms, consuming 49.1% calories during dark period. Surprisingly, HF-fed* CK1ε* ^*tau*/*tau*^ mice on a 20 hr LD cycle displayed improved diurnal rhythms of energy intake compared to the other groups on HF diet, consuming 64.6% of their total daily calories during the dark phase. No significant differences in absolute beam break activity levels (Figure 2(c)) and the activity patterns (Figure S2) were evident between wild-type mice and mutants on both RC and HF diet. Additionally, no differences in body temperature between genotype groups were observed under each diet condition (Figure S3).

### 3.3. Altered Fasting Glucose and Insulin Levels in* CK1ε* Mutants

**Figure 3:**
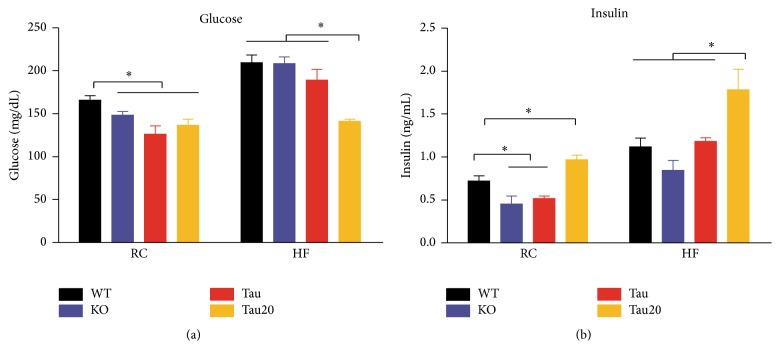
Altered glucose and insulin levels in* CK1ε* mutant mice. (a) Fasting serum glucose of mice fed either RC or HF diet. (b) Fasting serum insulin of mice fed either RC or HF diet.* CK1ε* ^*+/+*^ (white),* CK1ε* ^−/−^ (blue), and* CK1ε* ^*tau*/*tau*^ (red) mice were on a 24 hr LD cycle;* CK1ε* ^*tau*/*tau*^ mice represented in yellow were on a 20 hr LD cycle. Mean values are presented for each group, with error bars representing SEM. Asterisks indicate significant differences between groups (*p* < 0.05).

We then examined fasting blood glucose and serum insulin levels in samples collected during the light phase. On the RC diet, both* CK1ε* ^−/−^ and* CK1ε* ^*tau*/*tau*^ mice on a 24 hr LD cycle, as well as* CK1ε* ^*tau*/*tau*^ mice on 20 hr LD cycle, had slight, but significant, reductions in fasting glucose compared to wild-type mice (Figure 3(a);* CK1ε* ^*+/+*^ = 165.7 ± 5.4 mg/dL,* CK1ε* ^−/−^ = 148.3 ± 4.3 mg/dL,* CK1ε* ^*tau*/*tau*^ = 126.4 ± 9.3 mg/dL, and* CK1ε* ^*tau*/*tau*^ on 20 hr LD = 136.6 ± 7.1 mg/dL). On the HF diet,* CK1ε* ^*+/+*^,* CK1ε* ^−/−^, and* CK1ε* ^*tau*/*tau*^ mice on a 24 hr LD cycle exhibited increased fasting glucose levels that did not significantly differ from one another.* CK1ε* ^*tau*/*tau*^ mutant mice on a 20 hr LD cycle exhibited reduced fasting glucose levels compared to wild-types (Figure 3(a);* CK1ε* ^*+/+*^ = 209.4 ± 8.8 mg/dL and* CK1ε* ^*tau*/*tau*^ on 20 hr LD = 141.1 ± 2.5 mg/dL).

Complex changes in fasting insulin levels were also observed. As shown in Figure 3(b), on RC diet and a 24 hr LD cycle, both* CK1ε* ^−/−^ and* CK1ε* ^*tau*/*tau*^ mice had lower levels of insulin than wild-type mice, whereas* CK1ε* ^*tau*/*tau*^ mice on a 20 hr LD cycle exhibited higher fasting insulin levels than wild-type mice (*CK1ε* ^*+/+*^ = 0.72 ± 0.06 ng/mL,* CK1ε* ^−/−^ = 0.45 ± 0.09 ng/mL,* CK1ε* ^*tau*/*tau*^ = 0.52 ± 0.03 ng/mL, and* CK1ε* ^*tau*/*tau*^ on 20 hr LD = 0.97 ± 0.05 ng/mL). On HF diet, both* CK1ε* ^−/−^ and* CK1ε* ^*tau*/*tau*^ mice on a 24 hr LD cycle exhibited similar insulin levels. However,* CK1ε* ^*tau*/*tau*^ mice on 20 hr LD cycle exhibited a higher insulin level than wild-type mice on a 24 hr LD cycle (Figure 3(b);* CK1ε* ^*+/+*^ = 1.12 ± 0.10 ng/mL and* CK1ε* ^*tau*/*tau*^ on 20 hr LD = 1.78 ± 0.24 ng/mL).

### 3.4. Altered Glucose Tolerance in* CK1ε* Mutants

**Figure 4:**
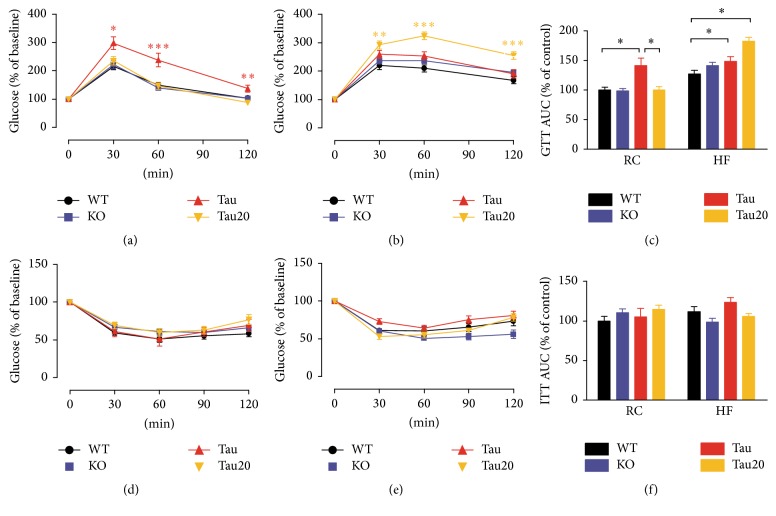
Alterations in GTT in* CK1ε* mutant mice. Top panel: GTT. Intraperitoneal GTT was performed in mice fed either RC (a) or HF (b) diet. (c) Area under the curve (AUC) of the GTT performed in (a) and (b). Bottom panel: ITT. Intraperitoneal ITT was performed in mice fed either RC (d) or HF (e) diet. (f) Area under the curve (AUC) of the ITT performed in (d) and (e).* CK1ε* ^*+/+*^ (white),* CK1ε* ^−/−^ (blue), and* CK1ε* ^*tau*/*tau*^ (red) mice were on a 24 hr LD cycle;* CK1ε* ^*tau*/*tau*^ mice represented in yellow were kept on a 20 hr LD cycle. Mean values are presented for each group, with error bars representing SEM. Asterisks denote significant differences between mutant genotype and wild-type controls on the same diet (^*∗*^ *p* < 0.05; ^*∗∗*^ *p* < 0.01; ^*∗∗∗*^ *p* < 0.001).

To evaluate glucose utilization in the mutant mice, we performed both a glucose tolerance test and an insulin tolerance test. Because mice of different genotypes had different baseline levels, the data presented here were normalized by dividing the observed glucose value from the basal level of each genotype under each diet condition. On RC diet,* CK1ε* ^*tau*/*tau*^ mice on a 24 hr LD cycle had a slower rate of glucose uptake than the other groups (Figure 4(a)), and the area under the curve (AUC) was higher in* CK1ε* ^*tau*/*tau*^ mice than* CK1ε* ^*+/+*^ mice (Figure 4(c);* CK1ε* ^*+/+*^ = 100.0 ± 4.9% and* CK1ε* ^*tau*/*tau*^ = 141.8 ± 11.7%).

On HF diet, mice of all genotypes exhibited a reduced rate of glucose uptake compared to mice on RC diet (Figure 4(c)), and, among the groups,* CK1ε* ^*tau*/*tau*^ mice on a 24 hr LD cycle were slightly slower than wild-type mice, and* CK1ε* ^*tau*/*tau*^ mice on a 20 hr LD cycle were the most altered, having a significant and sustained reduction in glucose clearance (Figure 4(c);* CK1ε* ^+/+^ = 126.7 ± 6.9%,* CK1ε* ^*tau*/*tau*^ = 148.5 ± 7.5%, and* CK1ε* ^*tau*/*tau*^ on 20 hr LD = 182.6 ± 6.0%). No significant differences were observed under either diet condition from mice of any genotype during the ITT (Figures 4(d)–4(f)).

## 4. Discussion

Using two genetic mouse models of* CK1ε* disruption (i.e., knock-out* CK1ε* ^−/−^ null mice and knock-in* CK1ε* ^*tau*/*tau*^ mutant mice) under different diet conditions, we have demonstrated distinct effects of the circadian clock gene* CK1ε* on body weight regulation and susceptibility to excess weight gain induced by HF diet. In particular, by maintaining* CK1ε* ^*tau*/*tau*^ mice on a 20 hr LD cycle, which more closely corresponds to their endogenous circadian period length and enables normal entrainment, we have generated evidence suggesting that proper entrainment and synchrony between internal circadian rhythms and the external environment may limit, or even protect against, the development of excess body weight gain induced by HF diet.

We found that both homozygous null and* tau* mutant mice had a reduced body weight, approximately 15% lower than wild-type mice on RC diet. The magnitude was close to the percentage (18%) of reduced body mass reported in* tau* hamsters. But we believe that the body weight change in each group of* CK1ε* mutants on RC diet is not caused by an accelerated circadian rate constant over physiological processes due to the discrepancy between impacts on body mass and circadian rhythms phenotype in* CK1ε* ^−/−^ and* CK1ε* ^*tau*/*tau*^ mice. We also noted that altered energy expenditure or intake does not appear to be the primary reason for the reduced body weight in* CK1ε* mutant mice, because we did not observe increased activity levels or reduced food intake in mutants compared to the wild-type mice. We also did not observe increased body temperature in mutants compared to the wild-type mice, which is consistent with previous results [20].

The cause of the reduced body weight in* CK1ε* mutant mice is still unclear, but there are some possible mechanisms worth testing in the future. First of all, higher metabolic rates may be the main factor to determine the low body mass, which have been shown in both homozygous* tau* mutant hamsters and mice [19–21], although no study has been done in the* CK1ε* null mutant. Second, it should not be excluded that alterations in development and cell growth might also contribute to the low body weight in* CK1ε* mutants, if considering the slow-growth phenotype in yeast with a deletion of a* CK1ε* homolog gene [23], as well as the known function of* CK1ε* in promoting cell growth [24, 25]. Therefore, a further analysis of pathways involved in cell growth, such as Wnt and its intracellular effector *β*-catenin, in* CK1ε* ^−/−^ and* CK1ε* ^*tau*/*tau*^ mice will help in testing this hypothesis.

Studies have consistently demonstrated that misalignment of feeding behavior and circadian rhythms or a disrupted circadian clock can cause altered body weight regulation and result in the development of abnormalities consistent with the metabolic syndrome [10, 11, 26, 27]. In particular, a recent study demonstrated the harmful effects of chronic circadian disruption on metabolism in wild-type mice [14]. The mice were housed on a 20 hr LD cycle, incongruous with their endogenous 24 hr circadian period, and displayed significantly increased weight gain after 6 weeks on the altered LD cycle. Complementing this previous study, we took a different approach by studying* CK1ε* ^*tau*/*tau*^ mice, which have an endogenous 20 hr circadian period, in both a 24 hr LD cycle and a 20 hr LD cycle. Remarkably, we observed that the endogenous circadian period matched 20 hr LD cycle protected against the HF diet-induced weight gain in* CK1ε* ^*tau*/*tau*^ mice. We found that HF-fed* CK1ε* ^*tau*/*tau*^ mice in a 24 hr LD cycle were no longer leaner than wild-types as the* tau* mutant mice were on RC diet, and many of their metabolic parameters, such as absolute body weight, body weight gain, gonadal fat pad mass, and fasting blood glucose, were similar to those of wild-type mice on HF. However, when the LD cycle was adjusted to match their shortened endogenous circadian period, all these parameters were restored toward levels of wild-type mice on RC diet. Additionally, although the diurnal rhythms of food intake in HF-fed mice of all the genotypes were attenuated, which is similar to what was shown previously [28], HF-fed* tau* mice on a 20 hr LD cycle displayed the least attenuation in diurnal rhythms of energy intake. However, unlike the altered body weight in wild-type mice on the shortened LD cycle [14], we did not observe any difference in body weight in* CK1ε* ^*tau*/*tau*^ mice on RC diet between the 24 hr LD cycle and endogenous circadian period matched 20 hr LD cycle. The different responses to RC and HF diets in* tau* mice may be due to increased sensitivity to diet-induced weight gain on a metabolically “challenging” HF diet, compared to the RC diet, in the* CK1ε* ^*tau*/*tau*^ mice. Another possibility is that we only monitored body weight in* CK1ε* ^*tau*/*tau*^ mice in a 20 hr LD cycle for 6 weeks during adulthood; we do not know if a longer exposure to an endogenous circadian period matched LD cycle or if rearing in 20 hr LD cycle from birth would restore the body weight in RC-fed* CK1ε* ^*tau*/*tau*^ mice. Further experiments are needed to address these questions.

It has recently been reported that* CK1ε tau* mutant hamsters are protected against the development of cardiomyopathy and renal disease by adjusting the environmental LD cycle to match their shortened endogenous circadian period [29]. Both the present study and previous experiments [14, 29] have demonstrated that certain metabolic and pathological abnormalities may be restored or prevented by optimizing the LD cycle and suggest that strategies designed to synchronize and match internal circadian cycles with the external environment may be useful in limiting or preventing the development of metabolic abnormalities. Synchronizing internal circadian cycles with the external environment has at least two beneficial effects on circadian organization: proper entrainment and resonance between environmental and internal circadian period length, which need not be mutually exclusive. The present study could not distinguish between these two effects, and future work utilizing entrainment-specific or period-specific mutants would be necessary to do so.

In the present study, we had some discrepant observations. For example, HF-fed* CK1ε* ^*tau*/*tau*^ mice on a 20 hr LD cycle had significantly lower body weight, but higher energy intake. This might be an interacting effect of a higher metabolic rate and a restoration of the LD cycles matching the endogenous period on the HF diet-induced weight gain. An improved alignment of the endogenous rhythms with the environmental LD cycle may improve the temporal coordination between feeding and metabolism, expending the energy intake at the correct time more efficiently. High metabolic rate alone or correct LD cycle alone may not act as effectively as in HF-fed* CK1ε* ^*tau*/*tau*^ mice on a 20 hr LD cycle. Another discrepancy in the results is that the HF-fed* CK1ε* ^*tau*/*tau*^ mice on a 20 hr LD cycle have lower fasting glucose but higher AUG in GTT. Although in many cases a reduced glucose level is associated with improved GTT, or a high level of glucose is associated with impaired GTT, a coexistence of both low glucose level and impaired GTT sometimes happens. One example is gene pancreatic-derived factor (PANDER), which was recently found to be a novel hormone regulating glucose levels via interaction with both the liver and the endocrine pancreas. Although still glucose intolerant, PANDER-deficient mice fed a HF diet are protected from HF diet-induced hyperglycemia because of the decreased expression of the gluconeogenic genes PEPCK and G6Pase and the reduced glucose production in the liver [30]. Although it is still unclear what the exact mechanism is for HF-fed* CK1ε* ^*tau*/*tau*^ mice on a 20 hr LD cycle showing both low glucose level and GTT intolerance in the present study, it is possible that the* tau* mutation affects certain gene(s) which function similarly as PANDER or its receptor.

## 5. Conclusions

In conclusion, we have demonstrated that differences in body weight regulation and the response to a HF diet challenge exist in two different mouse mutants of* CK1ε* on a 24 hr LD cycle, as well as* CK1ε* ^*tau*/*tau*^ mice on a 20 hr LD cycle that matches their endogenous circadian period. Both* CK1ε* ^−/−^ and* CK1ε* ^*tau*/*tau*^ mice had reduced body weights on RC diet despite similar overall caloric consumption and daily activity levels. On a HF diet, however,* CK1ε* ^*tau*/*tau*^ mice on a 20 hr LD cycle were protected against the development of excess body weight gain induced by HF diet. These findings may provide unique insights for future strategies of obesity management, which involve the nutrient composition of the diet, the properties and principles of the circadian clock system, and the interactions between these two factors in determining the metabolic responses.

## Supplementary Material

The supplementary material contains the growth curve of each genotype on RC in Figure S1, the beambreak activity profiles of each genotype on RC and HD in Figure S2, and the body temperature of each genotype on RC and HF in Figure S3.
