# Supplementary figures and images for: Altered Body Weight Regulation in CK1ε Null and tau Mutant Mice on Regular Chow and High Fat Diets
Source: Genet Res Int. 2016 Apr 6;2016:4973242. doi: 10.1155/2016/4973242 (PMC4837286; doi:10.1155/2016/4973242)

524 **Figure S1**

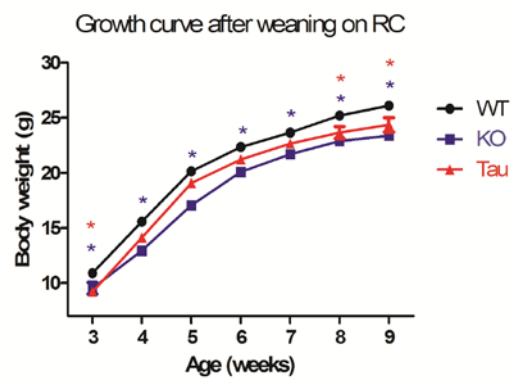

525

526

**Figure S2**

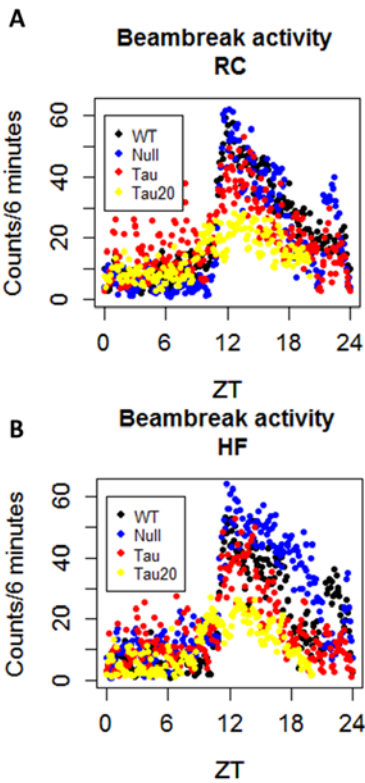

530 **Figure S3**

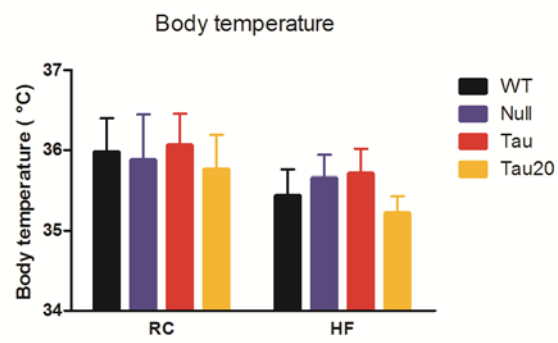

531

Supplement: Supplementary file 1 — The supplementary material contains the growth curve of each genotype on RC in Figure S1, the beambreak activity profiles of each genotype on RC and HD in Figure S2, and the body temperature of each genotype on RC and HF in Figure S3. [file 4973242.f1.pdf]
